# Racial Disparities Associated with the Prevalence of Vaccine and Non-Vaccine HPV Types and Multiple HPV Infections between Asia and Africa: A Systematic Review and Meta-Analysis

**DOI:** 10.31557/APJCP.2021.22.9.2729

**Published:** 2021-09

**Authors:** Jude Ogechukwu Okoye, Chiemeka Franklin Chukwukelu, Simon Imakwu Okekpa, Samuel Ifedioranma Ogenyi, Ifeoma Nora Onyekachi-Umah, Anthony Ajuluchukwu Ngokere

**Affiliations:** 1 *Department of Medical Laboratory Science, Faculty of Health Sciences and Technology, Nnamdi Azikiwe University, Nnewi Campus, Anambra State, Nigeria. *; 2 *Medbury Medical Services, Warri, Nigeria. *; 3 *Oncological and Radiological Sciences Cluster, Advanced Medical and Dental Institute, Universiti Sains Malaysia, Pulau Pinang Malaysia. *; 4 *Department of Medical Laboratory Science, Faculty of Health Sciences, Ebonyi State University, Abakaliki, Ebonyi State, Nigeria. *; 5 *Deworm the World Initiative, Evidence Action, Abuja, Nigeria. *

**Keywords:** Cervical Cancer, Human Papillomavirus genotypes, Vaccine, Africa and Asia

## Abstract

**Background/Objective:**

Cervical Cancer is the 6th most common and 3rd most deadly cancer among women. Despite the fact that the majority of the countries in Asia and Africa have a similar economy and low life expectancy, the mean age-standardized incidence rate (ASIR) of cervical cancer is substantially higher in Africa than in Asia. This study identified the correlates of the higher ASIR rates in Africa relative to Asia against two timelines; 2004-2009 and 2010-2017.

**Methods:**

Peer-reviewed articles published between 2004 and 2017 were selected using the PRISMA standard. Sources of articles included Google Scholar, Scopus, PubMed Central, and EMBASE. Search keywords included: HPV genotypes, cervical cancer, HPV vaccine, and multiple infections in Africa and Asia.

**Result:**

Twenty-nine and seventeen full-length articles were selected from Africa and Asia, respectively. The pooled prevalence of HPV infection up to 2017 was higher in Africa (41.8%; 95% CI: 35.9, 47.7) than in Asia (24.2%; 95% CI: 16.22, 32.2) at p< 0.001. Between 2004-2009 and 2010-2017 timelines, the pooled prevalence of HPV infection decreased from 49.1% to 36.7% (OR’: 1.66, 95% CI: 1.51-1.80) in Africa and increased from 16.9% to 20.5% (OR’: 0.79, 95% CI: 0.71-0.86) in Asia. However, the pooled prevalence of multiple HPV infections and non-vaccine high-risk HPV infections were higher among African women diagnosed with cancer (30.9% and 5.2%) than their Asian counterparts (21.0% and 2.0%, respectively) at p< 0.001. Additionally, the pooled prevalence of the five most prevalent high-risk HPV types in Africa were HPV16 (35.3%), HPV52 (14.2%), HPV35 (12.4%), HPV18 (10.4%), and HPV58 (10.0%), while that of Asia were HPV16 (37.3%), HPV52 (16.2%), HPV58 (14.7%), HPV33 (7.4%) and HPV18 (7.2%).

**Conclusion:**

This study suggests that the higher prevalence of HPV, multiple HPV and non-vaccine HPV infections could be responsible for the higher ASIR in Africa than in Asia.

## Introduction

Cervical cancer is the 9^th^ most common cancer in world, and the 6^th^ most common and 3rd most deadly cancer among women (Fitzmaurice et al., 2019). Studies show a minor reduction in age standardized incidence rate (ASIR per 100,000) from 14.5 to 13.1 between 2017 and 2018, and a minor increase in the age standardized mortality rate (ASMR per 100,000) from 6.1 to 6.9 within the same period (Fitzmaurice et al., 2019; Arbyn et al., 2020). Despite the fact that majority of the countries in Asia and Africa have similar economy (less developed), low life expectancy and high mortality-to-incidence rate (Chen et al., 2017), the mean ASIR/ASMR of cervical cancer was higher in Africa (29.4/19.8) than in Asia (11.3/6.2) as of 2018 (Arbyn et al., 2020). The findings of Canfell et al., (2017) suggest that the high mortality rate among cervical cancer patients in Africa is associated with low access rate to treatment. However the reason for the high incidence rate of the disease in Africa remains unknown. As of 2018, the ASIR/ASMR varies: 43.1/20.0, 40.1/30.0, 29.6/23.0/, 26.8/21.1, and 7.2/5.1 in Southern, Eastern, Western, central, and Northern Africa, respectively (Arbyn et al., 2020). This shows that Northern Africa had the least ASIR/ASMR in Africa, yet these values are higher than that of Western Asia, the least affected Asian sub-region (4.1/2.5) (Arbyn et al., 2020). Of note also, the ratio of cervical cancer attributable to HPV in Sub-Saharan Africa and Northern Africa/Western Asia is 9.3:1 (de Martel et al., 2017). Abryn et al. and Martel et al. attributed the low ASIR in Northern Africa and Western Asia to low prevalence of HPV. They did not offer any reasons for the variation in ASIR of cervical cancer and prevalence. We however hypothesize that this could be related to the peculiar HPV subtypes found in Africa and the type of vaccines adopted in the African sub-regions.

Commonly used HPV vaccines include bivalent (HPV16/18), quadrivalent (HPV6/11/16/18) and nonavalent vaccines (Gardasil 9; 6/11/16/18/31/33/45/52/58) (Center for Disease Control and Prevention, 2019). The first two vaccines are widely used in Africa but the nonavalent vaccine which has a global estimated efficacy of 87-89.5% in prevention of cervical cancer worldwide, is yet to be widely adopted in Africa (de Martel et al., 2017; Muñoz et al., 2003). Barriers to implementation of national vaccination programs in African countries include inadequate infrastructure and ﬁnances, limited health worker training, vaccine cost, and cold chain capacity constraints (Black et al., 2018). Even if nonavalent vaccine is widely distributed in Africa, majority of the countries in sub-Saharan Africa would not reach HPV elimination by vaccination alone. This is because, if HPV vaccination were to eradicate HPV types 6, 11, 16, 18, 31, 33, 45, 52, and 58, the incidence of cervical cancer will still be > 4/100,000 due to other non-vaccine high risk HPV (hrHPV) types that are not currently covered (Brisson et al., 2020). Furthermore, the effectiveness of this vaccine will depend on the rate of HIV infection in the target population which in turn drives multiple HPV infection. In Asia, the prevalence of multiple HPV infection in normal and abnormal cervix is 5.1-25.2% and 10.6-33.3%, respectively (Muderris et al., 2019; Ge et al., 2019; Al-Lawati et al., 2020) whereas in Africa, this is 3.9%-15.8% and 22.9-35.7%, respectively (Keita et al., 2009; Piras et al., 2011; Ndizeye et al., 2019). This underscores the fact that multiple HPV infection limits the efficacy of available vaccine to prevent the development of cervical cancer. This review aimed at identifying the prevalent HPV types in Asia and Africa both in the general population and among women with cervical abnormalities. It also assesses the paradigm shift in the prevalence of HPV infection in Africa and Asia between two timelines; 2004-2009 and 2010-2017. It determined the prevalence of HPV infection preventable by available vaccines.

## Materials and Methods

**Figure 1:**
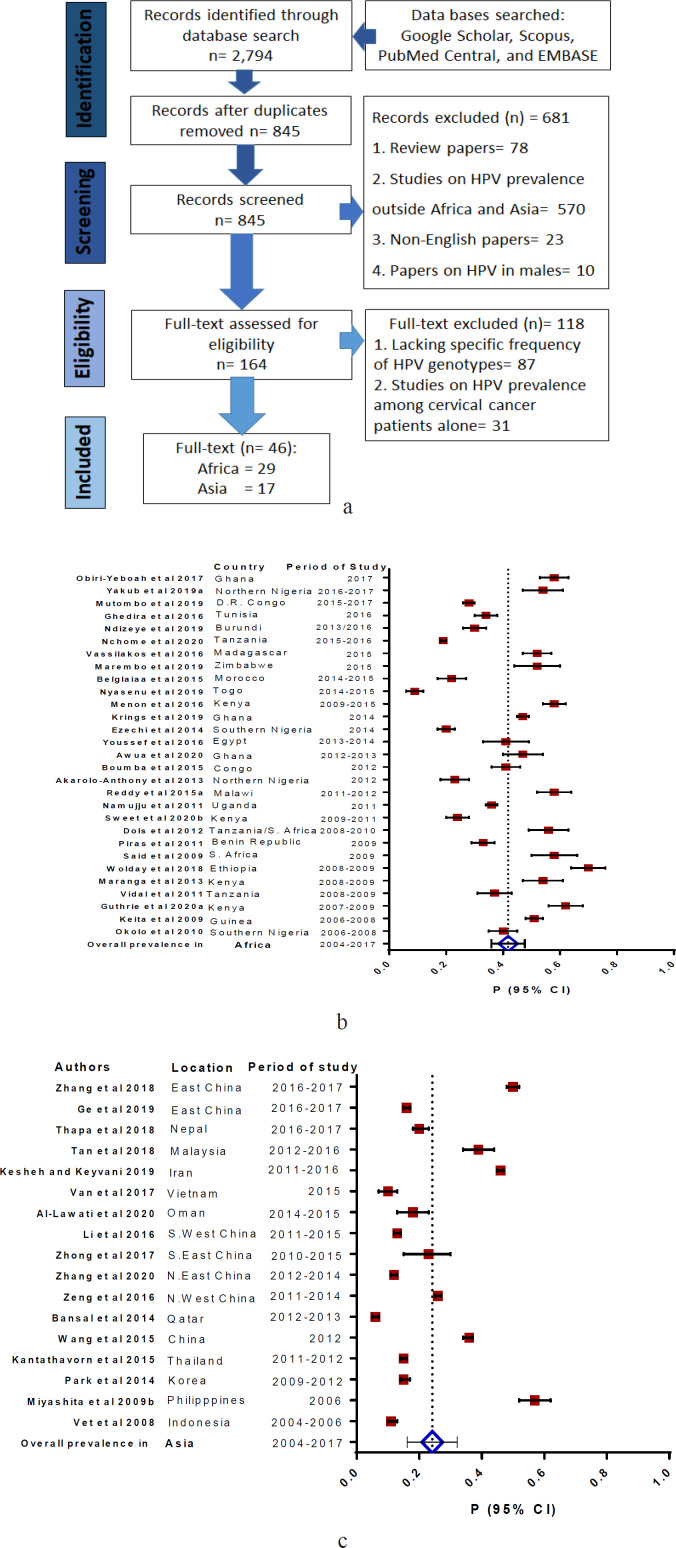
PRISMA Flow Chart Diagram of Study Selection. Figure 1b, Forest plot of pooled prevalence of HPV infection in Africa; Figure 1c, Forest plot of pooled prevalence of HPV infection in Asia. Figures 1b and 1c, The pooled prevalence of HPV infection up to 2017 was higher in Africa (41.8%; 95% CI: 35.9, 47.7) than in Asia (24.2%; 95% CI: 16.2, 32.2) at p< 0.001

Peer-reviewed articles published in Africa (n= 29) and Asia (n= 17) between 2004 and 2017 were selected and screened using the PRISMA standard (Figure1) (Liberati et al., 2009; Moher et al., 2009).

*Literature searches and Data sources*

Sources of articles include Google Scholar, Scopus, PubMed Central, and EMBASE. Search keywords included “prevalence or frequency of HPV types among women Asia AND Africa”, “distribution of HPV types among women with and without cervical cancer in Africa and Asia”, “vaccine AND non-vaccine HPV types in Africa AND Asia”, cervical cancer attributed to HPV infection in Africa and Asia”, and cervical cancer related mortality in Africa and Asia. Titles of cohort and case-controlled studies published between 2000 and 2019 were searched for using keywords and mesh terms: (‘HPV’ and ‘human papillomavirus’) AND (‘ICC’ and ‘Invasive cervical cancer’) AND (‘prevalence’ OR ‘incidence’ OR ‘distribution’ OR ‘genotype’), AND (‘Africa’), AND (‘Asia’). We also searched for unpublished studies (grey literature) by evaluating ClinicalTrials.gov (NIH), and International Clinical Trial Registry Platform (WHO). Inclusion criteria included: Studies with frequency of HPV infection, must be full-length articles and involve cervical cancer, and the articles must involve Africa and Asia. Exclusion criteria: Articles not written in English, abstracts, non-full-length article, and articles without specific frequency of HPV types, articles not involving Africa and Asia as well as articles not involving cervical cancer. 

*Data collection and extraction*

The vital information extracted for analysis included: participant characteristics such as sample size, cases of among African and Asian women, prevalence of any HPV infection and multiple HPV infection, HPV types (16, 18, 31, 33, 35, 39, 45, 51, 52, 53, 56, 58, 59, 66, 68, 82, 6 and 11), mean age, recruitment method, period of data collection, and study location and region (according to WHO classification). We investigated the frequency of HPV infection in between the two continents. When calculating the prevalence of any HPV infection (women who tested positive for any HPV type), an individual may have acquired both HPV35 and HPV45 or more but it would only count as one event. The range of high-risk HPVs investigated in the selected studies varied, thus when calculating the prevalence of an HPV type, only studies or cases that investigated that particular HPV type were considered. To assess the impact of timelines on the prevalence of HPV infection, data points were categorized into pre-and up to 2009, and from 2010 and up to 2017.

*Data analyses*

Pooled prevalence of HPV prevalence were presented in forest plots. Chi-square (*X*^2^) and Odds ratio analyses were used in calculating the difference in HPV types between Africa and Asia against different timelines (in GraphPad Prism, version 6.0). Significance was set at p< 0.05

## Results

Based on the inclusion criteria and according to World Health Organization classification of geographic regions, West Africa had the highest number of participants (7,313; n= 14 studies), followed by East Africa (7,240; n= 8 studies), Central Africa (2,167; n= 2 studies), North Africa (855, n= 3 studies), South Africa (153; n= 1 study). One mixed study involving Tanzania and South Africa (with 194 participants) was also included. Figures 1b and 1c show the pooled prevalence of any HPV infection in Africa and Asia, respectively. Studies in Africa revealed that HPV16, HPV18 and HPV52 ranked first/second in 44.8%/17.2% (13/29; 5/29), 10.3%/10.3% (3/29) and 13.8%/10.3% (4/29; 3/29), respectively whereas studies in Asia revealed that they ranked first/second in 70.6%/23.5% (12/17; 4/17), 0.0%/11.8% (0/17; 2/17), and 23.5%/17.6% (4/17; 3/17), respectively. Interestingly, HPV35 ranked first and second in 17% (5/29) and 20.7% (6/29) in studies in Africa but it only ranked fifth in one study from Asia (5.9%; 1/17, Tables 1a and 1b). Higher prevalence of HPV and multiple infections were observed in Africa than in Asia, both in the general population and among women with cervical abnormalities (p< 0.001; Tables 2-4; see supplementary and supporting data). Between 2004-2009 and 2010-2017 timelines, the pooled prevalence of HPV infection decreased from 49.1% to 36.7% (OR’: 1.66, 95% CI: 1.51-1.80) in Africa and increased from 16.9% to 20.5% (OR’: 0.79, 95% CI: 0.71-0.86).

*Prevalence of HPV types in multiple infection*

**Figure 2:**
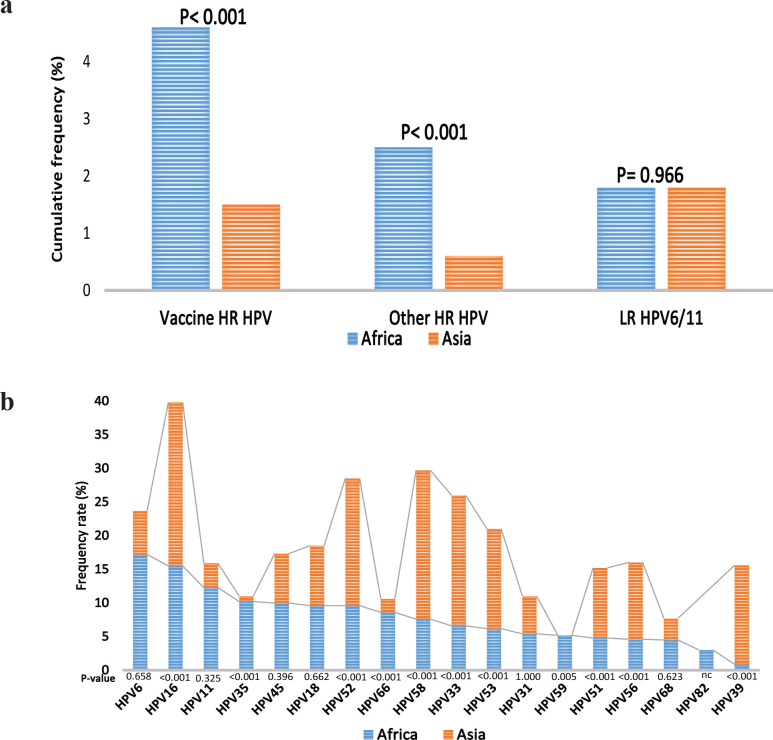
Frequency of HPV Genotypes in the General Population of Africa and Asia. Figure 2a, Higher prevalence of vaccine and non-vaccine HPV types were found in African than in Asia (p< 0.001) while similar prevalence of lrHPV types was found in Africa and Asia (p> 0.05). Figure 2b, In terms of multiple infection, significant differences were observed between Africa and Asia with regards to HPV58, HPV39, HPV33, HPV35, HPV52, HPV16, HPV53, HPV56, HPV66, HPV51, HPV59, HPV45, HPV68, HPV18 and HPV31, in descending order of rank. No comparison (nc) was carried out in respect to HPV82 due to the fact it was not investigated in the selected Asian studies used for plotting this graph

Considering the general population, significant differences in the prevalence of HPV types were observed between Africa and Asia, except for HPV6 (p< 0.001). High differences were observed between the two continents with regards to HPV16, HPV35, HPV18, HPV31 and HPV53/66 prevalence, in descending order of rank (Table 2). There were no observed differences between African and Asian studies with respect to vaccine hrHPV (p> 0.05) whereas, significant differences were observed between African and Asian studies with respect to non-vaccine hrHPV (p< 0.001). lrHPV showed no differences between African and Asian studies (p> 0.05; Figure 2a). The first five HPV types with the highest involvement in multiple infection in Africa were HPV6, HPV16, HPV11, HPV35 and HPV45 while that of Asia were HPV16, HPV58, HPV33, HPV52 and HPV53, in order of descending rank (Figure 2b). 

*Prevalence of HPV types among women with cervical abnormalities*

The prevalent HPV types in Africa were HPV16, HPV52, HPV35, HPV18 and HPV58 while the prevalent HPV types in Asia were HPV16, HPV52, HPV58, HPV33, and HPV53, in descending order of rank. High significant differences were observed between Africa and Asia with regards to HPV35, HPV6, HPV45, HPV31, HPV58, HPV66 and HPV68, in descending order of rank (Table 3). Result showed a higher prevalence of hrHPV and lrHPV in Africa than in Asia (p< 0.001; Figure 3Ia). It also shows that nonavalent HPV vaccine could prevent the development of 69.3% and 83.2% of HPV attributable cervical abnormalities in Africa and Asia, respectively whereas bivalent and quadrivalent vaccines could prevent the development of about 30% and 40% of HPV associated cervical abnormalities in Africa and Asia, respectively (Figure 3Ib). Cervical abnormalities among Africa women were 68%, 51%, 14%, and 67% more associated with multiple HPV infections, HPV18, HPV38 and HPV59 than the cervical abnormalities found among Asian women, respectively (Figure 3II). 

*Difference in HPV prevalence between 2004-2009 and 2010-2017*

Overall, the prevalence of HPV infection in Africa decreased between 2004-2009 and 2010-2017 whereas it increased in Asia between the same periods. Up to 2009, the pooled prevalence of HPV and multiple HPV infections were significantly higher in Africa (49.1% and 32.4%) than in Asia (16.9% and 5.1%, respectively) at p< 0.001. Up to 2009, there were significant differences between Africa and Asia with regards to the prevalence of HPV types in (p≤ 0.01), except for HPV-82 (p> 0.05). Within the same period, the prevalence of vaccine and non-vaccine HPV infection were higher in Africa (5.9% and 3.3%) than in Asia (1.1% and 0.7%, respectively). As of 2010 and later, the pooled prevalence of HPV infection and multiple HPV infection were significantly higher in Africa (36.7% and 14.3%) than in Asia (20.5% and 5.3%, respectively) at p< 0.001. This reveal that the multiple HPV infection decreased by 18.1% in Africa and increased by 0.3% in Africa between the timelines of 2004-2009 to 2010-2017 (Table 4; see supplementary data). No significant difference was observed between the two continents in terms of the prevalence of HPV6 and HPV11 (p> 0.05 and p< 0.05), respectively (Table 4). As of 2010 and later, the prevalence of vaccine and non-vaccine HPV were higher in Africa (5% and 2.5%) than in Asia (1.7% and 0.7%, respectively) at p< 0.001 (Figure 3c). Both in Africa and Asia, the prevalence of HPV16, HPV56, HPV51, HPV39 and HPV45 decreased from 2004 to 2017 (5% versus 16.0%, 1.5% versus 0.3%, 1.3% versus 0.1%, 0.7% versus 0.1%, and 0.6% versus 0.7%, respectively) while the prevalence of HPV53, HPV31, HPV68 and HPV33 increased (1.1% versus 0.2%, 0.9% versus 0.2%, and 0.3% versus 0.8%, and 0.2% versus 0.6%, respectively) from 2004 to 2017. This suggests that among hrHPVs the prevalence of HPV16 decreased the most whereas HPV53 increased the most. In Africa, the prevalence of HPV35, HPV59, HPV58, and HPV52 decreased 2.4%, 2.0%, 1.8%, and 0.4% from 2004-2009 to 2010-2017 while the prevalence of HPV66 and HPV18 increased by 1.3%, and 0.3%, respectively. In Asia, the prevalence of HPV82 and HPV18/66 decreased from 2004-2009 to 2010-2017 by 1.1% and 0.4%, respectively. Conversely, the prevalence of HPV58 and HPV52 increased by 1.5%, and 0.4%, respectively while the prevalence of HPV35 and HPV59 remained unchanged (Table 4; see supplementary data). In Africa, the risk of HPV infection and multiple HPV infections were 66% and approximately 3 times higher in ≤2009 than in ≥2010, respectively whereas in Asia, the risk of HPV infection and multiple HPV infections were 11% and 3% lower in ≤2009 than in ≥2010, respectively (Figure 4I).

*Prevalence of HPV types in the African sub-regions*

The highest and lowest prevalence of HPV infection as well as highest and lowest multiple infections were found in South Africa and Central Africa respectively. South Africa had the highest prevalence of HPV18, HPV45, HPV58, HPV35, HPV39, HPV51, and HPV59 while West Africa had the highest prevalence of HPV52, HPV53, HPV56, HPV66, and HPV82. East Africa had the highest prevalence of HPV31, HPV33, and HPV11 while North Africa had the highest prevalence of HPV16 and HPV6. Central Africa had the highest prevalence of HPV68. North Africa had the lowest prevalence of HPV45, HPV52, HPV58, HPV35, HPV66, HPV68, and HPV82 (Table 5; supplementary data). The pool prevalence of HPV in North, East, Central, West and South Africa were 32.0%, 30.6%, 30.1%, 44.9% and 58.2%, respectively while the prevalence of multiple HPV infections were 9.1%, 24.4%, 3.5%, 20.0% and 26.1%, respectively (see supplementary data). Result showed that the prevalence of non-vaccine hrHPV types were lower in the North and East Africa (Figure 4IIa) while the prevalence of HPV preventable by nonavalent vaccine was higher in both regions than other African sub-regions (Figure 4IIb).

**Table 1a:** Specific Distribution of Studies, Timeline, Sample Size and Prevalence of Multiple HPV Infections in Africa

Autho r(s)	Location/Country	Duration of Study	Age range/Mean	Cases	Multiple	Order of Prevalence
			years	N	HPV	High-risk HPV
Obiri-Yeboah et al 2017	Ghana	2017	≤29-60+/44	329	113 (34.3)	35,58,52,18,56
Yakub et al 2019a	Northern Nigeria	2016-2017	20-50/NA	220	25 (11.4)	35,16,45,33,18
Mutombo et al 2019	D.R. Congo	2015-2017	25-60+/46	1846	54 (2.9)	56,52,53,35.45
Ghedira et al 2016	Tunisia	2016	17-73/NA	471	5 (1.1)	16,31,58,66,56
Ndizeye et al 2019	Burundi	2013/2016	17-65/38	600	64 (10.7)	16,18,33,58,31
Nchome et al 2020	Tanzania	2015-2016	25-60/NA	3416	NA	52,16,58,18,35
Vassilakos et al 2016	Madagascar	2015	30-65/NA	403	143 (35.4)	53,68,52,35,16/33
Marembo et al 2019	Zimbabwe	2015	18-83/39	136	26 (19.1)	18,52,16,58,51
Belglaiaa et al 2015	Morocco	2014-2015	18-76/NA	232	34 (14.7)	16,53,18,52,31/33/56
Nyasenu et al 2019	Togo	2014-2015	20-50+/NA	324	7 (2.2)	18,45,16,68,35/52/53
Menon et al 2016	Kenya	2009-2015	18-61/28	616	202 (32.8)	16,53,52,56,58
Krings et al 2019	Ghana	2014	18-65/31	1943	188 (9.7)	16,52,35,59,66
Ezechi et al 2014	Southern Nigeria	2014	18-81/NA	515	28 (5.4)	16,35,58,31,18/52
Youssef et al 2016	Egypt	2013-2014	20-40/29	152	39 (25.7)	16,18,31,58/59,45
Awua et al 2020	Ghana	2012-2013	15-65/NA	226	151 (66.8)	16,35,58,45,18
Boumba et al 2015	Congo	2012	16-72/43	321	21 (6.5)	16,33,18,31,35
Akarolo-Anthony et al 2013	Northern Nigeria	2012	18-45+/37	278	23 (8.3)	35,56,58,45/59,68
Reddy et al 2015a	Malawi	2011-2012	25-59/NA	294	NA	58,35,16,33/52,18
Namujju et al 2011	Uganda	2011	NA/23	1943	366 (18.8)	18,16,45,33,52
Sweet et al 2020b	Kenya	2009-2011	18-50/NA	348	14 (4.0)	52,31,16/51,35/45
Dols et al 2012	Tanzania/S. Africa	2008-2010	20-73/NA	194	NA	52,16,66,33/51,35
Piras et al 2011	Benin	2009	15-70/NA	427	57 (13.3)	59,35,16,18,58
Said et al 2009	S. Africa	2009	NA/NA	153	40 (26.1)	35,18,58,16,66
Wolday et al 2018	Ethiopia	2008-2009	18-55+/41	233	3 (1.3)	16,35/45,31,56
Maranga et al 2013	Kenya	2008-2009	27-68/35	224	37 (16.5)	56,53,52,59,58
Vidal et al 2011	Tanzania	2008-2009	NA/40	215	149 (69.3)	16,35,58,45,51
Guthrie et al 2020a	Kenya	2007-2009	18-50/NA	283	122 (43.1)	52,16/18,51,35
Keita et al 2009	Guinea	2006-2008	15-64/NA	831	386 (46.5)	16,45,52,33,35/58
Okolo et al 2010	Southern Nigeria	2004-2006	15-80/NA	313	73 (23.3)	16/35,31,56,58

Keys, AR; Age range, MA, mean age, Prev.; Prevalent, HR; high risk, NA; Not available, D.R.; Democratic Republic, S. Africa; South Africa, a; HIV infected, commercial Sex Workers

**Table 1b:** Specific Distribution of Studies, Timeline, Sample Size and Prevalence of Multiple HPV Infections in Asia

Author (s)	Location	Duration of Study	AR/MA	Cases	Multiple	Order of Prev.
				N	HPV	HR HPV
Zhang et al 2018	East China	2016-2017	18-96/44	2612	346 (13.2)	16,52,58.18,53
Ge et al 2019	East China	2016-2017	16-85/NA	65613	2242 (3.4)	16,58,52,53,39
Thapa et al 2018	Nepal	2016-2017	20-65/32	998	61 (6.1)	16,39,58,33,51
Tan et al 2018	Malaysia	2012-2016	28-77/49	394	13 (3.3)	16,18,58,33,31
Kesheh and Keyvani 2019	Iran	2011-2016	13-74/NA	8351	1620 (19.4)	16,53,52,51,66
Van et al 2017	Vietnam	2015	18-49/NA	400	14 (3.5)	16,18,58/59,35/51/52/56
Al-Lawati et al 2020	Oman	2014-2015	18-68/NA	258	11 (4.3)	82,68,18/53/56
Li et al 2016	SW China	2011-2015	17-84/36	28375	664 (2.3)	52,16,58,18,56
Zhong et al 2017	SE China	2010-2015	16-77/36	71435	5354 (7.5)	16,52,58,33,18
Zhang et al 2020	NE China	2012-2014	25-65/39	34587	676 (2.0)	16,58,52,53,33
Zeng et al 2016	NW China	2011-2014	<30-50+/NA	51345	3382 (6.6)	16,58,56,39,18
Bansal et al 2014	Qatar	2012-2013	16-84/41	3008	6 (0.2)	16,56,59,18/31/45
Wang et al 2015	China	2012	NA	9641	486 (5.0)	16,52,58,59,56
Kantathavorn et al 2015	Thailand	2011-2012	20-70/44	5906	NA	52,16,51,53/58
Park et al 2014	Korea	2009-2012	39-58/NA	1998	40 (2.0)	16,18,51,58,52
Miyashita et al 2009b	Philippines	2006	18-40/24	369	94 (25.5)	52,16,66,45,59
Vet et al 2008	Indonesia	2004-2006	15-70/NA	2686	63 (2.3)	52,16,18,39,51

Keys, AR; age range, MA; mean age, HR; high risk, NW; North-Western, SW; South Western, NE; North East, NA; Not available, b; Commercial Sex Workers

**Table 2:** Prevalence Comparison of HPV Genotypes between African and Asian population

Variables	Africa		Asia		Africa vs Asia		Africa vs Asia
	Cases (N)	HPV+ n1 (%)	Cases (N)	HPV+ n2 (%)	n1-n2 (Rank)	X2 (p)	Pooled OR (95% Cl)
Any HPV	17486	6541 (37.4)	287976	58364 (20.3)	17.1	<0.001	2.35 (2.27, 2.41)
Multiple	13582	2482 (18.3)	282070	16274 (5.7)	12.6	<0.001	3.65 (3.46, 3.82)
Vaccine HR							
HPV16	17486	1396 (8.0)	287976	9413 (3.2)	4.8 (1)	<0.001	2.57 (2.55, 2.57)
HPV18	17486	927 (5.3)	287976	2765 (1.0)	4.3 (3)	<0.001	5.77 (5.31, 6.23)
HPV31	17486	669 (3.8)	287976	1294 (0.4)	3.4 (4)	<0.001	8.81 (8.08, 9.68)
HPV33	17253	439 (2.5)	287976	1952 (0.7)	1.8 (9)	<0.001	3.77 (2.69, 5.21)
HPV45	17165	669 (3.9)	287976	534 (1.9)	2.0 (8)	<0.001	21.4 (19.11, 23.81)
HPV52	17165	857 (5.0)	287976	7686 (2.7)	2.3 (7)	<0.001	1.88 (1.75, 2.01)
HPV58	17486	641 (3.7)	287976	5878 (2.0)	1.7 (10)	<0.001	1.83 (1.68, 1.98)
Other HR							
HPV35	17486	801 (4.6)	287976	578 (0.2)	4.4 (2)	<0.001	23.87 (21.33, 26.58)
HPV39	17165	247 (1.4)	287976	2679 (0.9)	0.5 (16)	<0.001	1.53 (1.35, 1.75)
HPV51	16932	402 (2.4)	287976	2293 (0.8)	1.6 (12)	<0.001	3.03 (2.72, 3.39)
HPV53	11928	312 (2.6)	276270	2098 (0.8)	2.8 (5)	<0.001	3.51 (3.13, 3.97)
HPV56	17486	388 (2.2)	287976	2391 (0.8)	1.4 (14)	<0.001	2.71 (2.44, 3.03)
HPV59	16852	370 (2.2)	287976	1537 (0.5)	1.7 (10)	<0.001	4.18 (3.74, 4.66)
HPV66	12927	408 (3.2)	278668	1243 (0.4)	2.8 (5)	<0.001	7.27 (6.49, 8.08)
HPV68	16738	373 (2.2)	284968	1608 (0.6)	1.6 (12)	<0.001	4.02 (3.60, 4.48)
HPV82	9045	158 (1.7)	101345	607 (0.6)	1.1 (15)	<0.001	8.33 (6.96, 9.97)
LR Type							
HPV6	9265	186 (2.0)	180707	4188 (2.3)	0.3 (17)	0.057	0.86 (0.74, 1.00)
HPV11	8241	132 (1.6)	180707	2404 (1.3)	0.3 (17)	0.034	1.21 (1.01, 1.45)

**Table 3:** Frequency of HPV Genotype among Women with Abnormal Cervix

Variable	Africa		Asia		Africa vs Asia	
Abnormal	Cases	HPV+	Cases	HPV+	n1-n2 (Rank)	Chi-square (p)
Cervix	(N)	n1 (%)	(N)	n2 (%)		
Any HPV	1035	815 (78.7)	961	820 (85.3)	6.6	<0.001
Multiple	544	168 (30.9)	757	159 (21.0)	9.9	<0.001
Vaccine HR						
HPV16	1035	366 (35.3)	961	358 (37.3)	2.0 (15)	0.402
HPV18	1035	108 (10.4)	961	69 (7.2)	3.2 (9)	0.012
HPV31	1035	78 (7.5)	961	25 (2.6)	4.9 (4)	<0.001
HPV33	972	52 (5.3)	961	71 (7.4)	2.1 (14)	0.076
HPV45	1035	84 (8.1)	961	12 (1.2)	6.9 (3)	<0.001
HPV52	960	136 (14.2)	961	156 (16.2)	2.0 (15)	0.227
HPV58	1035	104 (10.0)	961	141 (14.7)	4.7 (5)	0.002
Other HR						
HPV35	960	119 (12.4)	961	15 (1.6)	10.8 (1)	<0.001
HPV39	960	25 (2.6)	961	22 (2.3)	0.3 (17)	0.661
HPV51	960	62 (6.5)	961	28 (2.9)	3.6 (8)	<0.001
HPV53	742	23 (3.1)	961	32 (3.3)	0.2 (18)	0.89
HPV56	972	47 (4.8)	961	16 (1.7)	3.1 (10)	<0.001
HPV59	960	33 (3.4)	961	20 (2.1)	2.3 (13)	0.072
HPV66	867	58 (6.7)	961	23 (2.3)	4.4 (6)	<0.001
HPV68	897	34 (3.8)	961	9 (0.9)	2.9 (11)	<0.001
HPV82	769	27 (3.5)	757	7 (0.9)	2.6 (12)	0.001
LR Type						
HPV6	245	20 (8.2)	757	9 (1.2)	7.0 (2)	<0.001
HPV11	308	16 (5.2)	757	10 (1.3)	3.9 (7)	0.001

**Figure 3I:**
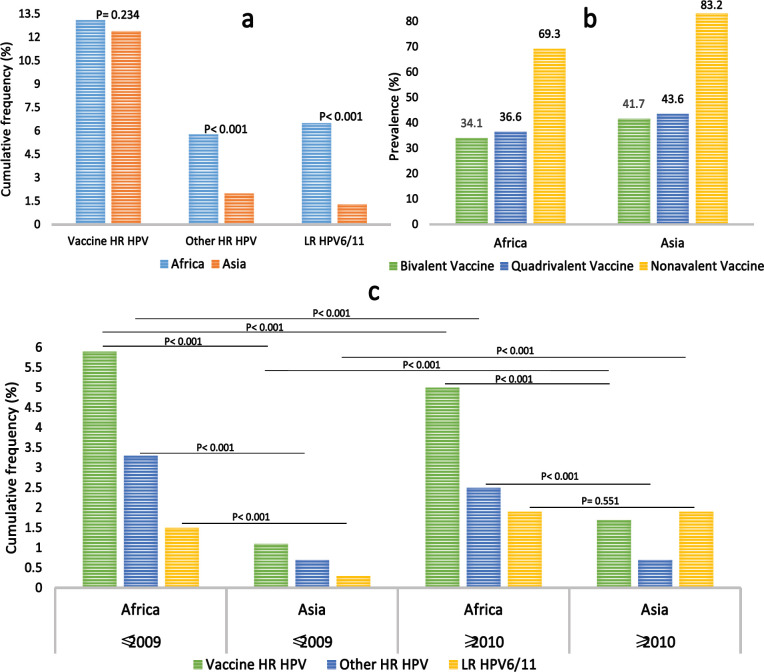
a) Frequency Comparison of HPV Genotypes in African and Asian women Irrespective of Cervical Status, b) Prevalence of HPV-associated cervical abnormalities preventable by available vaccines and c) Cumulative frequency comparison of vaccine and Non-vaccine HPV between Africa and Asia with regards to timelines. Figure 3I. No significant difference was observed between Africa and Asia studies in terms of vaccine hrHPV genotypes. However, significance differences were observed between Africa and Asian studies in terms of non-vaccine other hrHPV types and lrHPV16/11 (p< 0.001; Figure 3Ia). Figure 3Ib shows that quadrivalent vaccine may have mild edge over bivalent vaccine in preventing the development of cervical abnormalities both in Africa and Asia while nonavalent vaccine could exert almost twice the effect of bivalent and quadrivalent vaccines in forestalling the development of cervical abnormalities. Figure 3Ic, Cumulatively, as of 2009 and 2017, the prevalence of vaccine and non-vaccine hrHPV were significantly higher in Africa than in Asia (p< 0.001). As of 2009, the prevalence of lrHPV was significantly higher in Africa than in Asia (p< 0.001) while the prevalence of the viruses were similar between both Continents in 2017 at p> 0.05

**Figure 3II:**
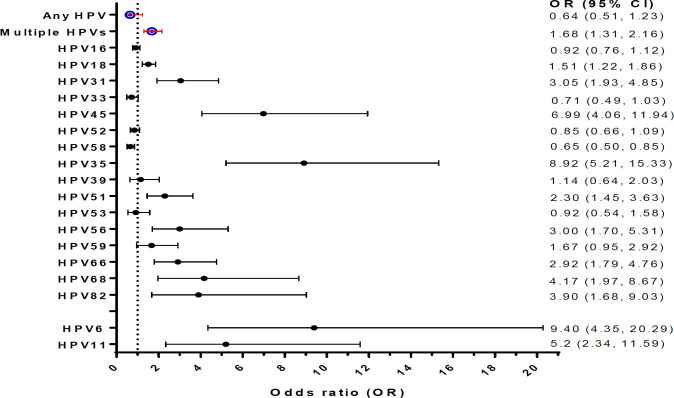
Meta-analysis of the Association between HPV Genotypes and Cancer (Africa versus Asia; derived from table 3). Figure 3II. Cervical abnormalities detected among Africa women were approximately 2 times, 3 times, 4 times, 5 times, 7 times, and 9 times more associated with HPV51, HPV-31/56, HPV-68/82, HPV11, HPV45, HPV-35/6 than the cervical abnormalities detected among Asian women, respectively

**Figure 4I:**
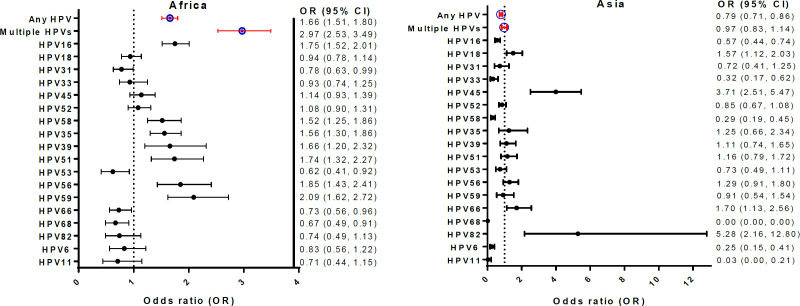
Prevalence Comparison of HPV Genotypes between ≤2009 and ≥ 2010 Timelines in Africa and Asia. Figure 4I, In Africa, the risk of HPV-52, 45, 58, 35, 39, 51, 16, 56, and 59 infections were 8%, 14%, 52%, 56%, 66%, 74%, 75%, 85%, and approximately 2 times higher in ≤2009 than in ≥2010, respectively whereas in Asia, the risk of HPV-39, 51, 35, 56, 18, 66, 45, and 82 were 11%, 16%, 25%, 29%, 57%, 70%, approximately 4 and 5 times in ≤2009 than in ≥2010, respectively. This suggests that in Africa the risk of HPV-6, 11, 18, 31, 33, 53, 66, 68, and 82 acquisition was higher in ≥2010 than in ≤2009, whereas the risk of HPV-6, 11, 16, 33, 52, 53, 58, 59, and 68 acquisition was higher in ≥2010 than in ≤2009

**Figure 4II:**
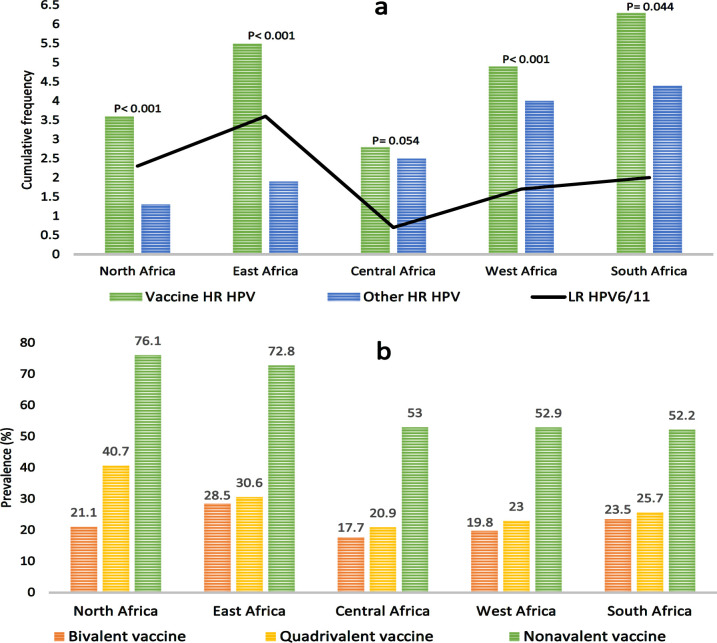
HPV Genotypes and HPV Preventable by HPV Vaccines based on African Sub-regions (see supplementary data). Figure 4a shows that the prevalence of non-vaccine (other) hrHPV infection is highest in South Africa but lowest In North Africa. It also shows that the prevalence of vaccine hrHPV infection and lrHPV infection was higher in South Africa and East Africa, respectively while the prevalence of vaccine hrHPV and lrHPV infections were lower in central Africa. Higher differences in prevalence of vaccine and non-vaccine hrHPV types were observed in North and East African sub-regions (p< 0.001). Figure 4b shows that the prevalence of HPV infection preventable by quadrivalent and nonavalent vaccines were higher in North and East Africa than in other African sub-regions

## Discussion

The incidence of cervical cancer in a population majorly depends on the prevalence of HPV infection and cervical cancer awareness, extent of high-risk sexual behaviour, prevalence of HPV infection and rate of HPV vaccination (Fitzmaurice et al., 2019; Arbyn et al., 2020; Klein et al., 2020). In Asia, although the rate of vaccination status is low (15.0%), knowledge of risk factors for cervical cancer and willingness to get vaccinated among females aged 19-23 years are high; 98.8% and 92.5%, respectively (Gollu and Gore, 2021). However, in Africa, HPV vaccine coverage in Africa (1.2-4.1%), the knowledge of risk factors for cervical cancer (7.8-11.8%) and willingness to get vaccinated (26.0%-58.8%) among females aged 16-20 years are all low (Ndikom and Oboh, 2017; Kifle et al., 2020). The low HPV vaccine coverage in Africa has been associated with high cost of vaccine and skepticism of its safety (Sopian et al., 2019). Considering the lack of implementation of World Health Organization (WHO) and Center for Disease control and prevention (CDCP)’s recommendations (World Health Organisation, 2014; Center for Disease Control and Prevention, 2019), a high ASIR of cervical cancer is expected in Africa (Arbyn et al., 2020; Klein et al., 2020). However, there is a need to indirectly assess the past and ongoing cervical cancer related interventions. There is also the need to identify major contributors to the high ASIR and ASMR in Africa for appropriate channeling of future interventions following recommendations. 

In the general population, this study revealed that HPV16, HPV52 and HPV58 were the most prevalent hrHPV types in Asia whereas HPV16, HPV18 and HPV52 were the most prevalent hrHPV infections in Africa. Considering the prevalence of hrHPV in abnormal cervix, this study revealed striking differences between Africa and Asia with regards to the prevalence of HPV35, HPV45, HPV31, HPV58, and HPV66. According to Hashim et al. (2020), the risk of developing CIN2+/CIN3+ among HPV16 and HPV18 positive women following a regular follow-up of 21 months are 24.4/19.9 and 16.2/10.8, respectively while the risk for women who are positive for other hrHPVs is 9.6/5.5. Based on the latter, the substantial higher prevalence of HPV16 and HPV18 in Africa relative to Asia might also be an explanation for the difference in cervical cancer ASIR between the two continents as revealed by Fitzmaurice et al. (2019) and Arbyn et al. (2020). Considering the fact that HPV16 infection is associated with high mortality among HPV positive women (Zhao et al., 2017), it could be argued that the high prevalence of HPV is linked to the high ASMR or poor prognosis among African women (Chen et al., 2017; Fitzmaurice et al., 2019; Arbyn et al., 2020). 

Namujju et al. (2011) stated that HPV16- and HPV18- positive women have 11-22-fold and 45-58 increased risk of acquiring other hrHPVs, respectively. Thus, the higher prevalence of multiple HPV infection observed in Africa relative to Asia could be due to the higher prevalence of HPV16 and HPV18 in African women. The multiple HPV infection could be responsible for the higher ASIR in Africa than in Asia since higher prevalence of multiple infection has been reported in abnormal cervix than in normal cervix (Keita et al., 2009; Piras et al., 2011; Ndizeye et al., 2019). In addition, multiple HPV infection has been linked to increasing prevalence of human immunodeficiency virus (HIV) which in turn contributes significantly to cervical carcinogenesis (Klein et al., 2020). Thus, it could be suggested that the high ASIR of cervical cancer in Africa when compared with Asia stems from a cascade of event beginning from non-vaccine HPV infection and high multiple infection. In this study, South Africa had the highest prevalence of any HPV and multiple HPV infection while Central Africa had the least of both parameters. The difference between the African sub-regions in terms of ASIR of cervical cancer as revealed by Martel et al., (2017) could be due to high multiple HPV infections and HIV infection rate (Hanischa et al., 2013; Taku et al., 2020). 

According to Huh et al. (2017), nonavalent vaccine prevents 90% of cervical cancer worldwide. Their estimate is substantially higher than the estimate of this study (69%). This could be attributed to their study not including any participants from African countries. Cervical cancers could be attributed to a single hrHPV type or multiple types such as HPV16, HPV18, HPV31, HPV33, HPV45, HPV52, and HPV58 which are preventable by available vaccines (de Martel et al., 2017). However, the vaccine hrHPV and non-vaccine hrHPV may not be effective in preventing multiple infection mediated cervical cancer involving HPV16/35, HPV16/53, HPV16/66, except in the event of cross-protection. Pirek et al. (2015) stated that there is an increased rate of malignant transformation in women with normal cervix and multiple HPV infection (< 365days) than in women with normal cervix and single HPV infection, hence the need for continual screening, even among vaccinated women and immunocompromised women. Given the high rate of multiple infections in Africa, especially West and South Africa, nonavalent vaccine may offer better protection against cervical cancer than other vaccines. Additionally, the hrHPV type with the highest difference between African and Asian women diagnosed with cervical abnormalities was HPV35. Consequently, the high prevalence of HPV35 and its involvement in multiple infection may account for the higher ASIR in Africa than in Asia (Fitzmaurice et al., 2019; Arbyn et al. 2020). Sadly, none of the available vaccines offer protection against HPV35, the second most prevalent HPV types, which accounts for 12.4% of cervical abnormalities in Africa. Since HPV35 appears to be the major correlate of cervical carcinogenesis in Africa, continual Pap smear screening is recommended.

In this study, between 2004 and 2017, the prevalence of HPV infection and multiple infection decrease by 12.4% and 18.1% in Africa while they increased by 3.5% and 0.2% in Asia, respectively. The reason for the observed decrease in Africa could majorly be due to increasing awareness. On the other hand, considering the fact that the widely distributed vaccine in Africa are bivalent and quadrivalent vaccines (Muñoz et al., 2003; de Martel et al., 2017), the decrease could be attributed to HPV vaccination since the prevalence of HPV16 substantially decreased (5%). Despite the fact that the overall prevalence of HPV infection decreased between 2004 and 2017 in Africa, the prevalence of HPV 33, 18, 6, 11, 82, 68, 31, 53, and 66 increased by 0.2%, 0.3%, 0.4%, 0.5%, 0.5%, 0.8%, 0.9%, 1.1%, and 1.3%, respectively within the same period, majority of the HPV types (56%; 5/9) are not covered by available vaccines. This may severely impact on the prevalence of cervical cancer in Africa. The reason however for the increased prevalence of HPV in Asia from 2004-2017 is still unknown. Interestingly, in Asia and Africa, the prevalence of HPV16 decreased by 16% and 5%, respectively while the following HPV types increased by certain percentage in 2017: HPV31 (0.2% vs 0.9%), HPV53 (0.2% vs 1.1%), HPV33 (0.6% vs 0.2%), HPV68 (0.8% vs 0.7%), HPV11 (1.3% vs 0.5%), and HPV6 (1.7% vs 0.4%, respectively). Differentially, the prevalence of HPV 52 and 58 increased in Asia by 0.5%, and 1.5%, respectively while in Africa the prevalence of HPV 18, 82 and 66 increased by 0.3%, 0.5% and 1.3%, respectively (Table 4). Though the prevalence of HPV 6, 11, 31, 33, 53, and 68 increased in both continents, the findings of this study suggest that if both continents adopt only nonavalent vaccines, it would still take a longer time to eradicate or significantly reduce cervical cancer in Africa than it would in Asia. This reiterates the fact that cervical cancer screening using Pap smear should still be an integral part of preventive measure in order to eliminate the disease in Africa by 2090 (Brisson et al., 2020).

The prevalence of vaccine and non-vaccine hrHPV were higher in South Africa than in other African sub-region while the prevalence of vaccine and non-vaccine hrHPV were lowest in Central Africa and North Africa. This study suggests that majority, over 72%, of cervical cancer attributable to HPV in North and East Africa could be prevented by vaccine, especially by using quadrivalent and nonavalent vaccines. Since 7 out of 12 countries are currently providing HPV vaccination at no cost for girls in East Africa, there is a higher chance of eliminating cervical cancer in the sub-continent by 2090 (Brisson et al., 2020; Njuguna et al., 2020). The findings of this study show that though nonavalent vaccine could prevent approximately 52% of cervical cancer attributable to HPV in Central, Western and Southern Africa, a tangible number of cervical cancer will still be observed in the sub-regions due to high prevalence of non-vaccine hrHPV.

*Limitation*

No grey literature was identified following our searches. This may constitute a limitation in this study.

In conclusion, This study revealed that the disparity between African and Asian women with regards to ASIR could be linked to high-risk HPV infection and multiple HPV types. It suggests that nonavalent vaccination could prevent over 90% of the cervical abnormalities in Africa. 

## Author Contribution Statement

This review was carried out and approved in collaboration between all the authors. JOO and AAN conceptualized and developed the study protocol. JOO, AAN, CFC, INO, SIO and ISO identified records for full-text review and data extraction. JOO and AAN drafted the manuscript.

## Ethical approval

This study is not part of an approved student thesis. It is exempt from ethical review and approval, since the data that were reviewed and used for meta-analysis were retrieved and synthesized from studies published in indexed journals. Informed consents and ethical clearance were individually sought and obtained by authors of the included articles. Hence, ethical approval to conduct the study was not sought from any scientific bodies.

## Supplementary data

Available at: Racial disparities in the prevalence of vaccine and non-vaccine HPV types and multiple HPV infection between Asia and Africa: A systematic review. doi: https://doi.org/10.1101/2020.11.02.20224857

## Conflict of interest

The authors declare that they have no financial or personal relationships which may have inappropriately

## Conflict of interest

The authors declare that they have no financial or personal relationships which may have inappropriately influenced them in writing this article.
